# Les troubles neurologiques secondaires à une carence en vitamine B12: analyse de 29 cas

**DOI:** 10.11604/pamj.2019.32.108.17609

**Published:** 2019-03-08

**Authors:** Maha Ait Berri, Abdellah Taous, Tarik Boulahri, Imane Traibi, Abdelhadi Rouimi

**Affiliations:** 1 Service de Neurologie, Hôpital Militaire Moulay Ismaïl, Meknès, Maroc

**Keywords:** Vitamin B 12 deficiency, Biermer’s disease, neurological disorders, Carence en vitamine B 12, maladie de Biermer, troubles neurologiques

## Abstract

Les troubles neurologiques secondaires à une carence en vitamine B12 sont polymorphes et variés. Peu de travaux se sont intéressés à leur analyse aussi bien dans la population marocaine qu'africaine. L'objectif de notre étude est donc de décrire les aspects cliniques, paracliniques et évolutifs des manifestations neurologiques des patients atteints d'une carence en vitamine B12, au sein du Service de Neurologie de l'Hôpital Militaire Moulay Ismail de Meknès sur une durée de 18 ans (1999-2017). Il s'agissait de 06 femmes et 23 hommes âgés en moyenne de 57 ans. Le délai du diagnostic était de 3 mois. L'atteinte neurologique était révélatrice de la carence en vitamine B12 dans 100% des cas. Le taux moyen de l'hémoglobine était de 10g/dl, le volume globulaire moyen de 115 fl. Une mégaloblastose médullaire et une gastrite atrophique étaient présentes dans respectivement 95% et 90% des cas. La réalisation systématique d'électroencéphalographie (EEG), électromyogramme (EMG) et potentiel evoqué visuel (PEV), a retrouvé chez nos patients des neuropathies périphériques et optiques infracliniques. Sur le plan étiologique, il s'agissait d'une anémie de Biermer dans 20 cas. Sous vitamine B12 parentérale, l'évolution était favorable chez tous nos patients. A la lumière de ces résultats, nous insistons sur l'intérêt du diagnostic précoce en raison de la gravité potentielle de certains tableaux neuropsychiatriques et le traitement substitutif qui doit être rapide car il est le seul garant d'une évolution favorable de ces atteintes.

## Abstract (en)

Neurological disorders secondary to vitamin B12 deficiency are polymorphic and diverse. There have been very few studies conducted in the Moroccan as well as in the African population. This study aims to describe the clinical, paraclinical, evolutionary features of neurological manifestations in patients with vitamin B12 deficiency within the Department of Neurology at the Moulay Ismail Military Hospital, Meknes over a period of 18 years (1999-2017). The study involved 06 women and 23 men, with an average age of 57 years. The mean time between symptom onset and diagnosis was 3 months. Neurological manifestation was indicative of vitamin B12 deficiency in 100% of cases. The average hemoglobin level was 10 g/dl, the mean corpuscular volume was 115 fl. Medullary megaloblastosis and atrophic gastritis were found in 95% and 90% of cases respectively. Regular electromyography (EMG), electroencephalography (EEG) and visual evoked potentials (VEP) showed subclinical peripheral and optic neuropathies. In 20 cases they were caused by Biermer’s anemia. All the patients received parenteral Vitamin B12 with good outcome. These results demonstrate the importance of early diagnosis in patients with a potentially serious neuropsychiatric condition as well as of rapid substitution treatment which is the only therapeutic option to secure a good outcome.

## Introduction

La carence en vitamine B12 est une situation fréquente [1]. Ses étiologies sont multiples et dominées par la maladie de Biermer et le syndrome de non-dissociation de la vitamine B12 chez le sujet âgé [2]. Ce déficit est habituellement révélé par des troubles digestifs et des anomalies hématologiques. Néanmoins, les formes isolées et les formes inaugurales de troubles neurologiques ne sont pas rares [1]. La vitaminothérapie B12, classiquement administrée par voie parentérale apporte un bénéfice dont l'importance dépend certes du type de l'atteinte neurologique, mais surtout de la précocité de son instauration [2-4]. A notre connaissance, peu de travaux ont étudié les troubles neurologiques secondaires à la carence en vitamine B12 au Maroc [5, 6], nous nous sommes alors proposés à travers cette étude d'analyser les profils épidémiologiques, cliniques, paracliniques et évolutifs des patients hospitalisés au Service de Neurologie de l'Hôpital Militaire Moulay Ismail et présentant des troubles neurologiques en rapport avec un déficit en vitamines B12.

## Méthodes

Il s'agit d'une étude rétrospective fondée sur l'analyse des dossiers des patients présentant des troubles neurologiques par carence en vitamine B12. Cette étude a été menée au sein du Service de Neurologie de l'Hôpital Militaire Moulay Ismail de Meknès au Maroc, sur une durée de 18 ans entre janvier 1999 et décembre 2017. On a inclut tous les patients présentant des troubles neurologiques en rapport avec une carence en vitamine B12 confirmée par le dosage sérique (taux inférieur à 200 mg/l). Plusieurs paramètres ont été étudiés: l'âge, le sexe, les antécédents pathologiques, les signes cliniques en particulier neurologiques et les données de l'hémogramme, du myélogramme, du dosage sérique de la vitamine B12, des explorations neurophysiologiques ainsi que de l'imagerie par résonance magnétique (IRM) médullaire. Tous les patients ont reçu une supplémentation en vitamine B12 sous forme d'hydroxocobalamine en intramusculaire (IM), suivant un protocole de traitement d'attaque suivi d'un traitement d'entretien. Le traitement d'attaque était administré à la dose de 5000 µg/j d'hydroxocobalamine en IM pendant une semaine, puis 5000 µg/semaine pendant 1 mois. Le traitement d'entretien consistait en une injection de 5000 µg/mois à vie dans le cas de la maladie de Biermer et séquentielle pour les autres étiologies. Les résultats étaient exprimés en valeur absolue et pourcentage pour les variables qualitatives, en moyenne, écart-type et valeurs extrêmes pour les variables quantitatives.

## Résultats

**Caractéristiques démographiques:** nous avons retenu 29 dossiers de patients présentant des troubles neurologiques en rapport avec une carence en vitamine B12 confirmée par le dosage sérique (taux inférieur à 200 mg/l). Il s'agissait de 23 hommes et de 06 femmes. Le sexe-ratio H/F était de 3.8. Dans notre série, l'âge moyen de nos patients était de 57 ans ± 5 ans avec des extrêmes allant de 45 à 73 ans (Tableau 1).

**Tableau 1:** caractéristiques démographiques, cliniques et paracliniques des patients présentant des troubles neurologiques secondaires à la carence en vitamine B12

Caractéristiques	Nombre de patients	Pourcentage
**Total des patients**	29	100%
**Démographiques**		
**Age**		
Médiane	57 ans ± 5 ans	
Valeurs extrêmes	45 - 73 ans	
**Sexe**		
Hommes	23	79.3%
Femmes	6	20.7%
**Cliniques**		
**Délai diagnostique**		
Moyenne	3.5 ± 2.1 mois	
Valeurs extremes	15 jours à 2 ans	
**Tableaux neurologiques avant les explorations éléctrophysiologiques**		
Atteinte médullaire	13	44.8%
Atteinte périphérique	12	41.4%
Atteinte encéphalique	2	6.9%
Neuropathie optique	2	6.9%
**Paracliniques**		
**Hémoglobine**		
Moyenne	10.0 g/Dl	
Valeurs extremes	6.3 – 12.0 g/dL	
**Volume globulaire moyen**		
Moyenne	115.95 fL	
Valeurs extremes	107 – 127 fL	
Macrocytose et anisocytose	26	89.6%
Mégaloblastose	29	100%
Taux sérique de la vitamine B12		
Moyenne	85.56 pg/mL	
Valeurs extremes	25 – 157 pg/mL	
**Tableaux neurologiques après les explorations éléctrophysiologiques**		
Atteinte médullaire	13	44.8%
Atteinte périphérique	20	68.9%
Atteinte encéphalique	6	20.6%
Neuropathie optique	16	55.1%

**Caractéristiques cliniques:** le délai du diagnostic moyen des troubles neurologiques secondaires à la carence en vitamine B12 était de 3.5 ± 2.1 mois (15 jours à 2 ans). Chez tous nos patients, la carence en vitamine B12 a été révélée par des symptômes neurologiques qui étaient classés en 4 formes cliniques: atteinte médullaire dans 13 cas, neuropathie périphérique dans 12 cas, atteinte encéphalique et neuropathie optique dans 2 cas chacune (Tableau 1).

**Caractéristiques paracliniques:** l'anémie a été retrouvée chez 26 patients, soit 89.65% des cas, avec un taux moyen d'hémoglobine (Hb) de 10.0 g/dL (6.3-12 g/dL). Le volume globulaire moyen (VGM) était élevé (macrocytose) chez 26 patients avec un VGM moyen de 115.95 fL (107-127 fL). Le frottis sanguin a été réalisé chez tous les patients. Une macrocytose associée à une anisocytose a été retrouvée chez 26 cas (89.6%). La ponction sternale a montré une moelle riche associée à une mégaloblastose chez tous les cas. Le dosage sérique de la vitamine B12 était bas chez 27 cas avec un taux sérique moyen de 85.56 pg/mL (25-157 pg/mL). Le dosage sérique des folates était normal chez tous les patients. Le dosage de l'homocystéine n'a été pratiqué que chez deux patients avec des taux sériques à 23.2 µmol/l et 18.8 µmol/l. Le dosage des anticorps anti facteur intrinsèque (AC anti FI) et anti cellules pariétales (AC anti CP) a été réalisé chez 26 patients. Les AC anti FI et les AC anti CP étaient positifs dans 12 cas (Tableau 1). L'IRM médullaire a été réalisée chez 13 patients avec des tableaux d'atteinte neurologique centrale. Elle a objectivé, chez 07 patients soit 24.1% des cas, des anomalies de signal hypo-intense en T1 et hyper-intense en T2 au niveau des cordons postérieurs et latéraux de la moelle cervico-dorsale. L'électroencéphalogramme (EEG), les potentiels évoqués visuels (PEV) et l'électroneuromyogramme (ENMG) ont été réalisés chez tous nos patients indépendamment de la plainte initiale du patient (Tableau 1).

**Données étiologiques:** les étiologies de la carence en vitamine B12 dans notre série étaient dominées par la maladie de Biermer avec 20 cas soit 69.0% de l'ensemble des patients. Elle a été retenue sur l'existence d'une gastrite atrophique fundique compatible avec une origine auto-immune chez tous les cas, associée à la présence des AC anti FI et des AC anti CP dans 12 cas. Le syndrome de non dissociation de la vitamine B12 de ses protéines porteuses (SNDB12PP) était l'étiologie la plus probable dans 7 cas soit 24.1%. Un cas avait une gastrectomie subtotale non supplémentée. Aucune étiologie n'a été retenue chez un seul patient refusant la fibroscopie (Tableau 2).

**Tableau 2:** données étiologiques et évolutives des patients présentant des troubles neurologiques secondaires à la carence en vitamine B12

Données	Nombre de patients	Pourcentage (%)
**Total des patients**	29	100
**Etiologiques**		
Maladie de Biermer	20	69,0
SNDB12PP	7	24,0
Gastrectomie subtotale non supplémentée	1	3,5
Etiologie inconnue	1	3,5
**Evolutives sous vit B12 IV**		
**Délai de la crise réticulocytaire**		
Moyenne	8.2 ± 4.5 jours	
Valeurs extremes	5 à 21 jours	
**Manifestations neurologiques**		
Récupération complete	16	55,2
Récupération partielle	13	44,8

SNDB12PP: syndrome de non dissociation de la vitamine B12 de ses protéines porteuses; vit B12 : vitamine B12; IV : intraveineuse

**Evolution:** tous les patients avaient répondu au traitement par vitamine B12 injectable. Une crise réticulocytaire était obtenue au bout de 8.2 ± 4.5 jours en moyenne (5 à 21 jours). Les anomalies hématologiques ont été corrigées après un mois. Les manifestations neurologiques se sont améliorées chez tous les patients suivis: une récupération complète dans 16 cas soit 55,17% des patients et une récupération partielle dans les autres cas. Aucun effet secondaire de la vitamine B12 n'a été noté (Tableau 2).

## Discussion

Dans cette série, la moyenne d'âge des patients et le sexe ratio étaient superposables à ceux rapportés dans la majorité des publications [4-7]. Les signes neurologiques étaient inauguraux chez tous nos patients contre 15,3% seulement dans la série de Maamar [6]. Le délai diagnostique de nos malades était court par rapport aux autres séries [4-7]. Ceci est peut-être dû à la facilité d'accès aux soins dans notre structure hospitalière militaire. Les manifestations cliniques neurologiques rapportées dans notre étude étaient conformes à celles décrites habituellement dans la carence en vitamine B12. Toutefois, la réalisation des examens complémentaires systématiques (PEV, ENMG) a permis le diagnostic d'atteinte infra-clinique et la découverte d'une fréquente association de plusieurs tableaux neurologiques. En effet, on a diagnostiqué une neuropathie optique chez 55% de nos patients grâce à l'apport des PEV. De même, le taux des neuropathies périphériques est passé de 44% à 68% grâce à la réalisation systématique de l'ENMG et plus de 50% des cas avaient au moins 2 sites atteints. Cette constatation a été rapportée par plusieurs séries [8-13]. Mayer *et al*. ont diagnostiqué 9 atteintes asymptomatiques chez 53 patients ayant bénéficié d'un EMG [9]. Puri *et al*. ont mis en évidence 31 myéloneuropathies, 5 myélopathies isolées et 4 neuropathies isolées chez 40 patients grâce aux explorations électrophysiologiques [10]. La neuropathie périphérique était présente chez 20 de nos patients et était associée à une atteinte optique dans 8 cas alors que 8 patients avaient une atteinte optique isolée. Fine et Hallet ont décrit des anomalies des PEV avec accroissement de la P100 chez 2 des 3 patients asymptomatiques sur le plan visuel [10]. Troncoso *et al*. ont montré l'existence d'anomalies des PEV chez 3 de leurs patients qui étaient asymptomatiques [11]. Saperstein *et al*. ont rapporté que différents syndromes peuvent être associés chez un même patient et notamment les scléroses combinées de la moelle et les neuropathies dans environ 50% des cas [12]. L'aspect retrouvé à l'ENMG est un processus axonal longueur dépendant avec de rares cas de neuropathies démyélinisantes. L'IRM cérébro-médullaire n'a pas été réalisée chez tous les malades. Elle prend de l'intérêt dans les présentations neurologiques pures sans anomalies hématologiques ou sans baisse de la vitamine B12 (Tableau 3).

**Tableau 3:** comparaison des résultats de notre étude avec ceux des autres séries

Caractéristiques	Notre série	Loukili [4]	El Otmani [5]	Maamar [6]	Bouchal [7]
**Tableaux neurologiques**					
Atteinte médullaire	44%	8.1%	67%	42%	57%
Atteinte périphérique	68%	24.5%	45%	57%	34%
Atteinte encéphalique	20%	8.1%	3%	3.8%	20%
Neuropathie optique	55%	0%	0%	0%	3%
**Biologiques**					
Taux moyen d’Hb (g/dL)	10	10.2	-	6.2	9.5
Taux moyen de VGM (fL)	115	109	-	109	107
Taux sérique du vit B12 (pg/mL)	73	73	-	-	53
Mégaloblastose	85%	75%	-	100%	75%
**Etiologiques**					
Maladie de Biermer	70%	100%	63%	57%	100%
SNDB12PP	20%	0%	8%	30%	0%
Carence d’apport	5%	0%	25%	23%	0%
Etiologie inconnue	5%	0%	25%	23%	0%
Syndrome auto-immun multiple	20%	26%	-	15.3%	17%

Hb: Hémoglobine; VGM: Volume globulaire moyen; vit B12 : Vitamine B12; SNDB12PP: Syndrome de non dissociation de la vitamine B12 de ses protéines porteuses

Sur le plan biologique, on note une tendance vers le tableau classique de la carence en vitamine B12 avec une atteinte hématologique quasi complète. Chez les patients diagnostiqués précocement, c'est le dosage des précurseurs “homocystéine et acide méthylmalonique” qui a permis le diagnostic, en l'absence d'anémie ou de macrocytose et même d'une carence en vitamine B12. En effet Oh *et al*. ont rapporté que 50 % des patients ayant une carence en vitamine B12 peuvent avoir un taux sérique en cobalamines normal [8]. Les résultats de notre série concernant le diagnostic étiologique étaient similaires aux données de la littérature [4-7] (Tableau 3). Seul un traitement substitutif précoce garantit une récupération clinique totale. Le traitement de l'atteinte neurologique ne diffère pas du traitement des formes sans atteinte neurologique. Il n’existe pas de recommandations concernant les modalités de prescription. Dans notre série tous les patients étaient mis sous le protocole d'un traitement d'attaque de 5000 µg/jour pendant une semaine d'hydroxcobalamine en IM puis 5000 µg/ semaine pendant un mois, suivi d'un traitement d'entretien de 5000 µg/mois. L'efficacité du traitement par voie orale a été démontrée dans plusieurs études, notamment sur les atteintes hématologiques. En 2010, Andrès *et al*. ont confirmé l'efficacité et le bénéfice de la vitamine B12 orale à travers une revue de tous les articles du traitement oral de la vitamine B12, publiés entre 1990 et 2007 [14]. La voie orale n'a pas été prescrite dans notre série car c'est une voie de traitement récente qui n'a pas assez de recul et aussi en raison du caractère rétrospectif de cette étude qui a concerné une longue période de 18 ans où le concept de non-dissociation de la vitamine B12 n'était pas encore bien connu. La récupération neurologique semble liée essentiellement à la précocité du traitement de supplémentation. Sous substitution vitaminique, l'évolution des atteintes neurologiques est diversement appréciée dans la littérature [8-10]. L'évolution favorable de la majorité de nos cas a été garantie par la précocité diagnostique.

## Conclusion

Notre étude a permis de laisser voir le caractère polymorphe et non spécifique des symptômes neurologiques du déficit de la vitamine B12, ce qui rend difficile à établir le diagnostic positif, d'autant plus que l'atteinte hématologique peut être absente ou atypique. Elle a montré l'importance d'un interrogatoire minutieux et d'un examen clinique complet ainsi que l'intérêt du dosage biologique de l'homocystéine et de l'acide méthylmalonique et surtout des explorations électrophysiologiques systématiques qui mettent en évidence l'atteinte neurologique diffuse en cas de carence en vitamine B12. Nous proposons alors d'élargir le dosage des cobalamines à toute atteinte neurologique ou psychiatrique dont la cause n'a pas été mise en évidence. Nous suggérons également d'inclure le dosage de l'homocystéine et de l'acide méthylmalonique et les explorations électro-neurophysiologiques dans l'arsenal diagnostique.

### Etat des connaissances actuelles sur le sujet

- La carence en vitamine B12 est une situation fréquente;
- Ses étiologies sont multiples et dominées par la maladie de Biermer et le syndrome de non-dissociation de la vitamine B12 chez le sujet âgé;
- Ce déficit est habituellement révélé par des troubles digestifs et des anomalies hématologiques; néanmoins, les formes isolées et les formes inaugurales de troubles neurologiques ne sont pas rares.

### Contribution de notre étude à la connaissance

- Les symptômes neurologiques du déficit de la vitamine B12 ont un caractère polymorphe et non spécifique ce qui rend difficile à établir le diagnostic positif, d'autant plus que l'atteinte hématologique peut être absente ou atypique;
- Un interrogatoire minutieux, un examen clinique complet ainsi que le dosage biologique de l'homocystéine et de l'acide méthylmalonique et surtout la réalisation systématique des explorations électrophysiologiques sont essentiels pour mettre en évidence toutes les atteintes neurologiques secondaires à la carence en vitamine B12;
- Il est recommandé d'élargir le dosage des cobalamines à toute atteinte neurologique ou psychiatrique dont la cause n'a pas été mise en évidence et d'inclure le dosage de l'homocystéine et de l'acide méthylmalonique et les explorations électro-neurophysiologiques dans l'arsenal diagnostique.

## Conflits des intérêts

Les auteurs ne déclarent aucun conflit d'intérêts.
